# Regarding: “Uncertainty and risk of misleading conclusions: an umbrella review of the quality of the evidence for ankle arthroscopy”

**DOI:** 10.2340/17453674.2025.44757

**Published:** 2025-09-19

**Authors:** Ömer Levent Karadamar, Ali Murat Başak

**Affiliations:** Department of Orthopedics and Traumatology, Döşemealtı State Hospital, Antalya, Turkey; Department of Orthopedics and Traumatology, Gulhane Teaching and Research Hospital, Ankara, Turkey

*Sir*, – we read with great interest the article by Ponkilainen et al. (2025) entitled “*Uncertainty and risk of misleading conclusions: an umbrella review of the quality of the evidence for ankle arthroscopy*” published in *Acta Orthopaedica* [1]. The authors should be congratulated for addressing a timely and clinically relevant topic. Their umbrella review highlights the critically low methodological quality of current systematic reviews on ankle arthroscopy and emphasizes the urgent need for more reliable evidence to guide practice.

While we agree with the authors’ conclusion that randomized controlled trials (RCTs) are essential, we believe that several complementary strategies can also contribute to strengthening the evidence base. Pragmatic multicenter trials are key components in bridging the gap between controlled efficacy and real-world outcomes. Bretherton et al. (2024) demonstrated in a large pragmatic RCT that early weight-bearing after ankle fracture fixation is non-inferior to delayed protocols and offers important functional benefits for patients [2]. Such studies are invaluable in providing evidence directly applicable to daily clinical decision-making. Similarly, Hohenschurz-Schmidt et al. (2022) systematically reviewed pragmatic pain trials, underscoring their methodological strength in reflecting real-world effectiveness [3].

Second, registry-based and multicenter data offer important insights when high-quality RCTs are lacking. Sánchez-Correa et al. (2025) analyzed revision and reoperation rates across different total ankle arthroplasty prostheses, highlighting the utility of collaborative, multicenter registries in generating meaningful outcome data for surgical decision-making [4]. Such registry-driven evidence can complement RCTs and provide valuable long-term safety information.

Third, recent technological advances in ankle arthroscopy merit attention. Lim et al. (2025) compared arthroscopic reduction and fixation with open reduction for pediatric intra-articular ankle fractures and reported superior outcomes in the arthroscopic group [5]. Moreover, Mercer et al. (2022) demonstrated that nano-arthroscopy in an office setting can be safely and effectively applied to posterior ankle impingement, yielding significant functional improvement and patient satisfaction [6]. These innovations illustrate that while high-level evidence remains limited, novel minimally invasive approaches continue to expand the scope of ankle arthroscopy.

*In conclusion,* the umbrella review by Ponkilainen et al. makes an important contribution by highlighting the low methodological quality of the current literature. However, we believe that progress in this field should not rely solely on future RCTs. Rather, a combination of pragmatic clinical trials, multicenter registry-based research, and transparent evaluation of novel arthroscopic techniques may together provide a more robust and clinically relevant evidence base for ankle surgery.


**Ömer Levent Karadamar** *Department of Orthopedics and Traumatology, Döşemealtı State Hospital, Antalya, Turkey* *Email: leventkaradamar@gmail.com*
**Ali Murat Başak** *Department of Orthopedics and Traumatology, Gulhane Teaching and Research Hospital, Ankara, Turkey* *Email: muratalibasak@gmail.com*

